# The History of Lithotomy

**Published:** 1831

**Authors:** James M. Staughton

**Affiliations:** Professor of Surgery in Miami University


ART. V. *The History of Lithotmoy.*—By JAMES M. STAUGHTON, M. D., Professor of Surgery in Miami University.

THE subject of Lithotomy is interesting to every Medical practitioner, and in this view I have no apology to offer to the readers of the Western Medical Journal for the sketch of the various operations which I am about to offer.

In the early ages of the world, surgery was left by the ignorant pride of the physicians to the superintendance of illiterate men, frequently to slaves, or to persons whom the father of Medicine denominates *mercenaries*, day-laborers. These men always operated, in all cases that were presented to them, with that boldness which is ever the associate of the most profound ignorance. In many cases they are said to have succeeded, but their modes of operation are to us entirely unknown.

Physicians, to the dishonor of the profession, neglected this important province of the Healing Art, and seemed willing to commit every thing surgical to an abyss from which there should be no resurrection.

The history of the operation for Lithotomy, is shrouded in so thick a veil of antiquity, that though its existence is ascertained, the peculiarities of its form are not traceable. The first record that we have of this most distressing affliction, is found in the celebrated oath of Hippocrates, in which he makes his pupils swear "by Apollo Medicus, by Esculapius, and by Panace, and calls all the gods and goddesses to the oath, that they will never cut any one for the stone, but leave the operation to the surgeons."

The next author, in point of time, to whom we must refer is Celsus. He mentions a certain Ammomius as the most ancient lithotomist, and describes a kind of scoop and another instrument which he employed when operating. Meges is mentioned as a successor of Ammomius. He is said to have brought his instruments to a state of very considerable improvement. It is certain that Ammomius and Sostrates also, endeavored to break the calculus in pieces while yet in the bladder, and then to extract the fragments. Florus, the animated Latin historian, states that the son of Alexander, monarch of Syria, perished under the operation of lithotomy. The usurper of his father's kingdom pretended that he labored under calculus, and contrived his death during the pangs of incision. From this solitary fact, the state of surgery and the dissolute character of surgeons at that period may be readily inferred. It is a fact, that, as we sometimes say, speaks volumes.

Celsian Operation.—The first operation in lithotomy that we find distinctly described, is in the works of Celsus. The operation was probably performed long before the time before he flourished, but here we have the first distinctive exhibition. It has been called *Lithotomia Celsiana* and *Methodus Celsiana*, by a variety of authors, not because invented or performed by this celebrated writer, but because he was the first who described it.

About the commencement of the 16th century, the operation was called the APPARATUS MINOR, a name by which it is pretty generally known, and which distinguishes it from another mode, which was denominated apparatus major. Another name has been employed, more appropriate than elegant, called "THE CUTTING ON THE GRIPE," from the peculiar manner in which the stone was seized previous to the incision.

It was the opinion of the older surgeons, that a wound in the bladder itself was infinitely more dangerous than one in the neck of that viscus. Hence, it became an important object with them to extract the stone solely through an aperture produced in the neck. It is not to our present purpose to shew how completely they were mistaken. The patient was placed on the table, very nearly in the same position in which we place him now. The legs were secured by two assistants. The fore and middle fingers of the left hand of the surgeon, previously dipped in oil, were introduced into the anus; the right hand rested on the abdomen, just over the symphysis of the pubes. With the fingers of the left hand, attempts were made to draw the calculus down to the neck of the bladder and by these means to produce a tumour in the perineum, by which the incision was to be guided. But the appearance of this tumour afforded very uncertain direction. In none but in cases of very small calculi could it be produced. As soon as the tumour appeared, or in other words, when the calculus was supposed to be fairly lodged in the neck of the bladder, the incision was to be made. Celsus directs that it be made above the anus, of a semicircular form, and that the *extremities* of it should be below. The stone was then dislodged by the fingers in the rectum, or if it were too large, an instrument resembling the common obstetric crotchet was used to facilitate its exit. What I have described is the celebrated Celsian mode, a mode devised and practised in the infancy of Anatomy, yet one which exhibits some elemental knowledge in relation to the parts to which an operation in lithotomy has immediate relation.

For example, the semi-lunar cut was evidently intended to avoid wounding the rectum.

But there are many and very serious objections to this Celsian course. The operation when thus performed, must have occurred without that anatomical precision, with which it ought ever to be attended. In almost every case, the parts in which incisions are intended to be made, materially vary, corresponding with the magnitude of the stone, the force employed to keep it opposite to the perineum, and its precise situation as regards the bladder. The urethra may entirely be cut away from the prostate. The vesiculæ seminales, the vasa deferentia, or even the ureters themselves may be seriously injured.

According to Celsus himself, the neck of the bladder has been so much injured from the pressure of a rough calculus upon it, that patients have been destroyed from this cause alone. But the most formidable objection remains to be exhibited. The extreme difficulty of reaching the stone in adults, and of fixing it in an apposite situation for extraction was well known to the ancient, and, under this serious impression, Celsus permitted the operation to be performed only on patients between 10 and 14 years of age.

We have presented you undoubtedly with the most interesting of operations for one of the most deplorable of maladies; interesting as one of the first and finest efforts of chirurgic skill, interesting from its simplicity, and interesting because of the success which attended its performance. I consider it as an operation performed by some happy and adventurous hand, in the rude, primeval ages, without a parallel. We contemplate it with enthusiastic admiration, and wonder that a first attempt should have approximated so near to perfection. In relation to children, this simple mode of operating far from being disdained, has been advised by surgeons of the present day.

Celsus is the only writer who expounds the process with any thing like precision, and his successors have touched the subject only with a trembling hand. What they have written is remarkable only for its obscurity. Galen, though he writes expressly on the stone, and prescribes remedies, almost innumerable, calculated to dissolve it in the bladder, utters not a word relative to an operation.

Antylus, Paul of Ægena, and the celebrated Phylagreus, while they have thrown great light on the state of other branches of medical science, in the days in which they lived, have contributed nothing to the boundaries of our knowledge of Lithotomy.

From the point we have assumed, let us descend to that period when the Arabians, enriched with the spoils of European learning, transplanted the arts from their native soil to Asiatic shores. And what shall we find here? Among them Surgery never flourished. An appalling horror at the idea of operation paralysed the hand which held the surgical knife. Prejudices both of a civil and religious character fettered their genius, and restrained its daring aspirations. Life was trifled away with the ointment, the balsam, or the talisman, where the bistoury or the scalpel offered the only chance of salvation. In the hands of men thus tenacious of blood, lithotomy, as might be expected, could make no advances. Content with what Celsus had written, no effort at improvement was attempted. The infallibility of Celsus was acknowledged by the Arabians, as firmly and satisfactorily as by the Romans themselves. It is surprising, yet true, that Albucasis, the Hunter in Arabic Surgery, has left no indication of improvement on the Celsian mode, in even a solitary particular. On the contrary, the only alteration he ventured to make on the system of his great master's method, tends simply to show his want of firmness. This frightened operator, if during the course, hemorrhage occurred, would immediately apply green vitriol, and with a wisdom, at which modern surgery cannot forbear smiling, wait until the hemmorhage had subsided. To the Arabian, the lithotomist owes nothing, but gratitude, for their having preserved to us the illustrious records of Celsus.

High Operation.—The simple cutting on the gripe maintained its character, and was the only operation known for the calculus, until the commencement of the 16th century. Peter Franco, a celebrated surgeon of Lausanne, about this period had the honor of devising and of executing a new mode. The account which he has given of the circumstances which led him to this new idea, is so natural, that I shall content myself with translating his own words, from the antiquated French of his day. "I will mention," says he, "what once happened to me when desirous of extracting a stone from a child two years of age or thereabout. In this case I found the stone as near as might be to the size of a hen's egg. I did all that it was possible for me, to draw it out, but found I could not move it with all my efforts. The patient was extremely tormented, and the relations were desirous that the child should die, rather than remain in such unutterable affliction. To my shame be it spoken, I also was not willing to be reproached with not knowing how to extract the stone. With the consent of the father, mother, and friends, I determined to cut the child above the pubis. I made an incision above the penis, holding the stone with my fingers in the rectum, and having the abdomen compressed by an assistant. In this way I succeeded. The patient was cured (but not without severe indisposition) and the wound perfectly healed."

The distinguished lithotomist who thus communicated to the world the first conception of the high operation, was deeply tinctured with the prejudice which had been transmitted to himself and his cotemporaries from the writers of antiquity, that wounds in the body of the bladder were necessarily fatal. Notwithstanding his boldness in the preceding case was crowned with the most abundant success, his strong apprehensions as to wounds of this nature, prevented him from recommending the high operation to his followers. Another idea concerning the stone is to be found in the work of Franco, which deserves to be recorded, since within a very few years a distinguished surgeon in London has applied it to the most successful practice. In the case of females afflicted with the stone, he declines the use of the knife, confining himself to the employment of a peculiar instrument for dilating the urethra, and then extracting the stone with forceps.

In 1580, Francis Rousset alleged the success of Franco, as a ground for venturing to assert that the operation above the pubis might be performed without subjecting the patient to any greater risk than he would be exposed to, in the common mode of operating. Though Rousset never attempted the operation, he determined in a masterly manner the process by which it ought to be performed. As the high operation has been attempted after the instructions of Rousset, and in no other way until the appearance of Frere Come, a slight outline of it in this place, may not prove uninteresting.

Having distended the bladder in a great degree, either by injections of barley-water, or by the retained evacuation, the patient was laid upon a table with his hips a little raised. An incision was then made from the symphysis pubis towards the umbilicus, of the breadth of three or four fingers in extent, between the recti and pyrimidales muscles. The bladder being in this way exposed, a spear-pointed bistoury was plunged through its anterior walls, as near as possible to the cervix. This opening was to be small, to prevent the too sudden escape of urine, and to allow a probe-pointed knife to be introduced, by which the incision was to be enlarged, but not to be greater than should be found absolutely necessary for the introduction of the forceps. The forefinger of the left hand, then being pushed into the rectum, the stone was so raised that it could easily be picked out with the fingers, or by means of a small pair of forceps. The lips of the wound were then to be completely closed and healed as rapidly as possible.

Such was the operation of Rousset—an operation in which all chances of failure or accident were carefully provided against. But the dogmas of the ancients were not to be relinquished by the operators of that day, consequently the high operation fell into oblivion, until resuscitated by John Douglass, who, in 1719, placed it in the ranks of regular and established surgery.

His method so closely resembles that of Rousset, that a particular description of it is unnecessary. He demonstrated its excellency by experiment, as his brother, James Douglass, had previously shown by repeated anatomical examinations, that it was perfectly feasible. Out of fifteen patients who were cut by this method in Westminster and St. Thomas Hospitals, in London, all, with the exception of two, were perfectly restored. Yet, while the efficacy of Douglass's mode was, by surgeons, generally admitted and regarded in a light the most favorable, the unbounded success of Cheselden so attracted their attention, that the operation above the pubis became, in a good degree, disregarded.

After this, an ecclesiastic, commonly known under the name of Frere Come, attempted to revive this operation under a modified form, in France.

This operation soon fell into neglect. It has since been brought up again, by an irregular lithotomist of Paris, named Souberbielle, who claims some kind of a descent from Frere Come. In this operation a catheter is introduced into the bladder; the first incision of the lateral operation is made and carried down to the catheter in the membranous portion of the urethra; on the catheter is introduced into the distended bladder, a spear-pointed sound, the point of which is pushed from the bladder through the muscles and integument just above the pubis. This opening is then enlarged, and the stone extracted.

The first incision enables the urine to drip away at the most depending part. In the year 1823, the writer saw Souberbielle, in Paris and in London, where his operations were eminently successful. But they were frowned on by the regular surgeons.

In London, Souberbielle operated on several of Sir Astley Cooper's patients, with very marked success.

Apparatus Major.—The high operation not coming into use, the Celsian mode continued to be employed. The lithotomists of this period were men ignorant and avaricious; abhorred and rejected by the practitioners of medicine.

The latter, however finding that the operators had a highly lucrative branch of the profession under their care, attempted to divert it into their own hands. The Celsian mode now in use, was simple, and required nothing but intrepidity to ensure success. To invent an operation more complicated in its nature, and requiring more dexterity in its performance, and then to induce the world to believe that it was infinitely superior to the popular practice, became an object of invention and ambition. In this way they promised to secure to themselves those pecuniary rewards of which the lithotomists were reaping such an abundant harvest, and to suppress them beneath a mass of learned jargon and incomprehensible absurdities.

Johannes de Romanis, a surgeon of Cremona, in Italy, has merited the honor of having encumbered our science with that mode of extracting the stone, called the Great Apparatus. This projector, though he acquired while living, a degree of reputation which few men attain, has left no description of his invention. Mariano Santo de Barletta, a Neapolitan by birth, a disciple of Romanis, wrote extensively on the merits of his master. As his account of the Great Apparatus was the first given to the world, his name has been applied to it. It is called the Marian mode; just as the name of Americus is given to the continent of Columbus.

*The Marian operation* was called the *Apparatus Major*, on account of the number of instruments necessary for its performance, in distinction from the Celsian *Apparatus Minor*, where only a knife and a hook were required. A passage was to be selected from Hippocrates, on which this operation was to be considered as founded: for without some high authority, no practitioner would dare to hazard an innovation. An aphorism was therefore selected from the writings of this father of medicine, which long experience had decided to be false, but which, as it favored the ideas of Mariano, was supported with as much intemperate fervour, as though it were impossible for Hippocrates to have erred. The celebrated selection is "*vesica decissa, aut cerebro, aut corde est lethale,*" wounds of the bladder, of the brain, or of the heart, are mortal. This I say was known to be false, since in the apparatus minor, and in the high operation, the bladder had always been cut, and frequently been cured.

Let us attend for a moment to the process by which this operation was performed. The patient was placed on a table, and two strong bands of cloth, three yards long, joined in the middle, were placed behind the neck, two ends were brought over the shoulder and two under the armpits, when all four were laced across the thorax. The thighs were thin, violently flexed on the pelvis, and the legs on the thighs; the patient was then requested to grasp the soles of his feet with either hand. The straps were now brought down from the thorax under the thighs, and the patient lashed hand and foot together. Two assistants were employed to hold the legs, while a third was victoriously mounted on the table, behind the victim, to secure him the more effectually by holding him down. Nor was this precaution in having him thus pinioned, useless, as you will discover when we mention the horrible tortures to which he was about to be subjected.

———"Quis talia fando

Myrmidonum, Dolopumve aut duri miles Ulysses

Temperet a lachrymis."

The instruments necessary to this operation were, a common metallic catheter, a lithotome, which was an instrument bearing a strong resemblance to an abscess lancet set in a wooden handle. Two iron rods called conductors were employed, one, the male, furnished with a ridge along its whole length, the other, the female, was bent and grooved at its extremity. These, with forceps, were the most important instruments. But who shall describe the complexity of others that embarrassed the operation—the simple and compound dilators, the aperiens, the lateræ! Instruments well calculated to perplex the surgeon and to torture the patient, but not to extract the calculus.

With the lithotome, originally with a razor, the incision was made through the bulb of the urethra to the catheter, which had been previously introduced. A probe was then slipped in along the catheter to the bladder, then the male conductor was slipped in along the probe, and the female conductor slided along the male. The surgeon then seizing the projecting ends of the conductors, separated them in correspondence with the median line of the body. Thus commenced the process of dilatation, as it was denominated. This dilatation was nothing less than a violent laceration of the membranous portion of the urethra, the prostate gland, and the neck of the bladder. Can any operation be more dreadful than this laceration? Reflect on the exquisite sensibility of the urethra, and then conceive, for a moment only, the introduction of a probe or a catheter, and then the forcing of two iron rods into this delicate membrane. Look at the remorseless surgeon, with an unrelenting hand, stretching, jerking, and tearing this membrane, on which the finest sensibility has been conferred, for half an hour together. Think of the laceration of the prostate gland, and you cannot wonder that the poor sufferer was bound so firmly, or a monster placed behind him to press him to the table and drown his cries.

When the laceration (for dilation it was not) was supposed to have become sufficiently large, forceps were introduced, and the stone was seized. This grasping of the stone was the signal for a renewing of the tortures of the patient. The surgeon was generally fatigued before the operation was carried sufficiently far to allow the exit of the calculus. This, however, was of no consequence, as the dragging out of the stone would only be an increase of the tearing. When the forceps were found too weak to educe it, additional instruments, called lateræ, were applied round them, and then by increased power, the cause of the calamity was brought out. A silver canula was then introduced, and the canal was stuffed with lint and plugged with compresses.

That this barbarous operation, having in it more of the infernal than the human, could have taken the place of the simple Celsian section, must appear incredible to every one not acquainted with the ideas and feelings of the profession at the period of its introduction. It was an invention that the common lithotomists had neither the presumption nor the cruelty to attempt. The pride and pecuniary advantages attendant on inventions, frequently induce, as it induced the physicians of the age to which we are referring, to multiply writings in their vindication. These writings, which are of the most pompous descriptions, abounding with sesquipedalian words, were well calculated to produce a powerful effect on this half enlightened age.

The apparatus major, once in fashion, every surgeon found himself under the necessity of rendering the subject more complicated by the introduction of some new instruments. Fabricius, of Heldanus, that illustrious surgeon and philosopher, though he preferred Franco's mode, with a fanatacism that would have seemed inconsistent with the talents of a man so eminent, devised new instruments for this execrable grand apparatus. Like an official of the inquisition, he valued himself, we fear, on the discovery of new modes of torture. Alas for man! amid the multiplied calamities of his dejected condition, that the means of relief should be found more tremenduously formidable than the calamities themselves. We would hope that merely to torture could never have been the aim of any surgeon under the sun; but where *real ignorance* and *fancied science* are united, their offspring are "misery, lamentation, and woe." Under various modifications, the mode of operating which we have described continued predominant until the year of 1664. At this period a man named Rau appeared in Paris as a professional lithotomist. His was neither the high operation nor the grand apparatus. He adopted a novel mode. He made his external wound to correspond with the internal, in order that uriniary extravasation might not occur. For a long period his ability was distinguished.

*Lateral Operation.*—Some time after, a wandering lithotomist had the honor of presenting a new, but in his uninstructed hands, a very imperfect mode of operating for the stone. I allude to the celebrated Frere Jacques. His early life had been spent with one Paulon, a lithotomist, from whom he acquired the manipulation of cutting. He visited Paris, where, at first, he was well received, permitted to practise his art in the Hospital, and was presented to the King. His piety was ardent, his enthusiasm unbounded. Considering himself anointed of the Lord for this most important office, he operated with boldness, and his success was surprising. His method is thus described by Bussire.

"He never tied his patients. He introduced his staff, large, round, and having no groove, into the bladder. Holding it in his left hand, he pressed it so against the perineum as to make the part project, where he meant to strike. Then taking into his hand, a long knife, dagger-shaped, he plunged it in, fearlessly, near the left buttock, two inches from the perineum; pushing it onwards, he opened the body of the bladder as near as possible to the neck, and never once withdrew it until he had produced an orifice equal to the magnitude of the stone. He then introduced his finger and forceps, and drew out the stone, leaving the patient with the words "The operation is finished—*God will cure you.*"

As might be expected, such an operation, in the hands of an itinerant, perfectly ignorant of the structure of the parts, into which he unhesitatingly penetrated his pious knife, could not always be successful; and yet his success was great. If the powers of immagination can enlist with advantage any principle that can assist in recovery from disease, he certainly fixed on the noblest which human nature can conceive. Wholly disinterested, it was nevertheless his lot to meet persecution in its most violent forms. The whole circle of medical men rose in arms against him, and he was reduced to the necessity of leaving Paris. Some years afterward he returned. Fagon, the physician of the king, who at this time labored under the disease whose history we are tracing, persuaded him to study anatomy, and allured him to the use of the grooved staff, by means of which, his operations were rendered still more certain, and his success in consequence still more brilliant. Adopting this improved mode, he operated on thirty-eight patients, in succession, at Versailles; every one of whom recovered. This benevolent man travelled over a large part of Europe, pursuing his profession and receiving no compensation but what was requisite to repair his instruments and patch his shoes.

During the time he was in Holland, Rau, who was afterwards a professor in the University of Leyden, caught, as it is supposed, from him, the modus operandi. Rau, however, never revealed the peculiarities of his mode. Surgeons crowded to witness his operations; but to every solicitation for explanation, he would reply, "Celsum Legitote." It has been asserted that Rau cut 1540 patients, and that not one of them died. This has been considered exceedingly questionable.

"Credat Judæus Apelles—non ego."

After the death of Rau, which occurred in 1719, various experiments were made for the purpose of obtaining his mode of operating for lithotomy. Among these experimentalists was the celebrated Cheselden. After a series of trials, he became convinced that it was impossible to open the bladder, with safety, without striking through the membranous portion o the urethra and the prostate gland. On this he discontinued his investigations, and directed all his skill and practice to make the operation succeed. The experiments of Cheselden, previous to this determination, were called his first method. His second method was this. A grooved staff was introduced through the urethra. The skin and fat were pierced just as the operation is, in the present day, performed. The fore-finger was introduced into the upper angle of the wound, and the groove of the staff felt—the assistant then drew up the staff as high as possible under the arch of the pubis, that the knife might not touch the rectum. The membranous portion of the urethra, the prostate gland, and the neck of the bladder were then divided by pushing forward the knife. The left fore-finger being kept along the groove on the back of the knife. The beak of a blunt gorget was introduced into the groove, and urged on to the bladder—the staff was then withdrawn, and the forceps passed in along the concavity of the gorget. The third method which he practised afterwards, differed very little from the second. In the third, he cut the prostate more fully than he did in the former, while engaged in withdrawing his knife. While operating agreeably to the manner ascribed by

Albinus to Rau, he lost four patients out of ten, but in pursuing his own improved method, his success was most splendid. Of 52 patients on whom he successively operated, he lost but 2. In fact, of the whole number of persons whom he proceeded to relieve, and which consisted of 213, he lost only 20.

The mode of Cheselden, which we trace through Rau to Frere Jacques, and which was now called the lateral mode, acquired popularity abroad, and was illustrated and perfected at home, by Sharp and Broomfield.

About the middle of the 18th century, another itinerant Friar produced a considerable change in the French lithotomy. The invention of a new instrument was published in a French newspaper, and ascribed to a Friar, named Come. This instrument consisted in a curved bistoury about five inches long, which was concealed in a slender steel case, but from which it could be brought out by means of a spring, and the angle which it would form with the case could be estimated by a scale on the handle. Now in the operation of Frere Come, the first incision of Cheselden down to the grooved staff, was practised. The lithotome cache, which, when shut, was nothing more than a common probe, was slipped along the grooved staff into the bladder; then touching the spring, the blade appeared, and in drawing out the instrument, the neck of the blader, the prostate gland, and the membranous portion of the urethra were divided. This operation, slightly modified, prevailed universally in France, and is still employed in that scientific country.

The following extract from Tavernier's excellent work on operative surgery, will exhibit the manner in which this operation is performed at the present day.

"The lateral operation, which is at present almost exclusively adopted in France, consists in making an incision upon the left side of the perinæum, commencing about one inch in front of the anus, and extending as far as the middle of a straight line drawn from the anus to the tuberosity of the ischium, including the adipose cellular tissue which is contained in the space between the erector penis and accellerator urinæ muscles, the transversus perinæi, the anterior fibres of the levator ani, and the membranous part of the urethra, and in dividing from within outwards, according to the method of Frere Come, the neck of the bladder and the left lateral part of the prostate, by means of the instrument called the *lithotome cache.*—We shall now proceed to give an account of this operation as described by Professor Boyer.

*Apparatus.*—The instruments which are required for performing this operation are a silver catheter, and a grooved staff of as large a size as will easily admit of introduction; a lithotome cache; a sharp gorget; a large sclapel for making the first incision; a blunt-pointed curved bistoury; forceps of various sizes and forms, for extracting the stone; a syringe for washing out coagula of blood or particles of stone; several gum-elastic or silver canulæ; ligatures, and a pair of ligature forceps; two strong garters or bands, for tying the patient's hands and feet; a bowl of warm oil, and basins containing tepid water, and a decoction of marsh mallows.

*Preliminary arrangements.*—Before the operation is commenced, it is necessary to procure a firm table, of sufficient height, about three feet in width, and from four to five in length. This table is to be covered with a mattress, which is fastened with a rope, and arranged in such a manner as not to pass over the edge upon which the patient is to be placed; a pillow should then be laid at its upper end, and the mattress should be covered with a folded sheet, which is to be left hanging to within a foot of the floor. About an hour before the operation is commenced, care should be taken to shave the perinæum, and to empty the rectum by means of a clyster. This last precaution is of the utmost importance, and should never be neglected. It need scarcely be remarked here, that, a short time before the operation, the patient should be kept on low diet, take the warm bath, and drink plentifully of diluent drinks: he should also be desired to refrain from sleeping on the day on which the operation is to be performed, and if he be irritable, he should take about fifty drops of laudanum.—Five assistants are usually required in this operation, two for holding the thighs and legs of the patient, one for steadying the shoulders, and another for holding the sound and the scrotum, and the fifth for handing the instruments to the operator. In operating upon children, it is necessary to have another assistant to steady the pelvis, by placing his hands upon the spine of the ilium.

Having every thing ready, the patient is to be placed upon his back, his head is to be slightly elevated, and his thighs and legs are to be held by two assistants. The surgeon then takes the staff, first dipped in oil, and after having introduced it, and ascertained that the stone is really in the bladder, the instrument is to be given to an assistant standing on the left of the patient. Two strong garters or ligatures, each about two yards in length, are then to be doubled, and placed by means of a noose round the patient's wrists, who is next to take hold of his feet with his hands, in such a manner that the fingers shall be applied to the soles, and the thumbs upon the dorsal surface. The two ends of the garter are then to be carried in opposite directions round the ankle, over the back part of the hand, and under the foot, so as to form a kind of figure of 8; after which they are to be tied in a bow.

The ligature being thus applied at the same time on each side, the two assistants who are to hold the extremities, apply the patient's knees against their breasts with the right hand, while they take hold of the soles of their feet with the left. Another assistant, who stands behind the patient, applies his hands upon his shoulders to prevent him from moving back, while the one who hands the instruments is to stand on the left of the surgeon.

*Operation.—First stage.*—" Every thing being thus arranged, the operator, standing between the thighs of the patient, and a little towards the left side, places the staff in a direction perpendicular to the axis of the body, by inclining its handle towards the patient's right groin, and gives it to an assistant, who is to be requested to hold it perfectly steady. If the scrotum be small, he should hold it up with the ulnar edge of the left hand, and extend the skin of the perinæum transversely with the thumb and fore-finger but if it be large and pendulous, the assistant who holds the staff should keep it up with the left hand, taking care not to compress the testicles, and draw the skin of the perinæum from below upwards. The surgeon then takes a scalpel in his right hand, and holding it as in cutting from without inwards, he makes an incision through the skin and adipose cellular tissue on the left side of the perinæum, extending from the raphe, at about an inch above the anus, to the middle of a straight line drawn from the anus to the top of the tuberosity of the ischium." This incision should be from two to three inches in length, and extend merely through the skin, the adipose cellular tissue, some of the fibres of the erector penis and accelerator urinæ, the transversus perinæi and the anterior fibres of the levator ani muscles. It may be laid down as a general rule, that it should rather be too large than too small, in order to prevent urinary infiltrations. In corpulent individuals, this first incision does often not extend as far as the urethra, so that it becomes necessary to make it deeper. When it is finished, the surgeon has to introduce the left index-finger, in order to ascertain the situation of the sound and judge of the thickness of the parts by which it is covered. If this thickness be considerable, it will be requisite to extend the incision still deeper; after which the index-finger is to be disposed, in such a manner that its radial edge shall look downwards, and that the left edge of the groove of the staff shall be lodged in the hollow which separates the nail from the bulb of the finger. The operator then takes a straight bistoury, held like a writing pen, carries its point upon the nail, and passes it into the groove of the staff across the parietes of the urethra. When it has arrived there, which may always be known by the immediate contact of the two instruments, the extremity of the finger is to be applied upon the back of the bistoury, and while its handle is gently raised, its point is to be passed into the groove of the staff. The handle of the bistoury is then to be depressed, in order to make the instrument describe the segment of a circle round its point, which is to be kept firmly fixed, and to divide the part of the urethra by which it is covered. The incision of the urethra should be from eight to ten lines in length, and should extend, if possible, merely through the membranous portion. When this incision has been made, the surgeon takes the lithotome cache in his right hand, and passes its beak into the groove of the staff. Having done this, he takes hold of the handle of the latter instrument with his left hand, and brings it towards the arch of the pubes, at the same time that he pushes the lithotome from below upwards, in order that its beak may retain its situation in the groove. The lithotome being thus introduced into the bladder, its beak is to be gently struck against the staff, and after having ascertained that it is still in the groove, it is to be pushed in as far as the point where its extremity terminates in a cul-de-sac, at the same time that the handle of the staff is brought a little towards the surgeon. The latter instrument is then to be withdrawn, while the operator takes the lithotome at the junction of the blade and sheath with the thumb and fore-finger of the left hand, and holding it in a position parallel to the external wound, he disengages the cutting blade of the instrument from its sheath. The surgeon is then to draw the lithotome towards himself, in a perfectly horizontal direction, so as to make the requisite division of the prostate gland and neck of the bladder.

"The great art of conducting the lithotome," observes M. Boyer, "consists in giving it a perfectly horizontal direction, and in carrying the edge of the blade parallel to the incision in the integuments. If the handle of the instrument be elevated, there will be danger of wounding the bas-fond of the bladder; if it be depressed, the incision through the prostate gland and neck of the bladder will not be proportioned to the degree of the opening of the blade; if the blade of the instrument be directed too far outwards, there will be danger of injuring the inferior, and, perhaps, the deep-seated branch of the internal pudic artery; and if the edge be carried too far down, it will be likely to interfere with the rectum." In order to avoid these inconveniences, this surgeon has advised, in operating upon adults and old people, never to open the blade of the lithotome beyond No. 11, and in most cases not farther than No. 9, preferring to enlarge the incision, if this be found necessary, to make it too large in the first instance. M. Boyer is also in the habit of applying the beak of the lithotome against the neck of the bladder, instead of letting it rest against the arch of the pubes, and of holding it in a transverse direction. After the prostate gland and neck of the bladder have been divided to the requisite extent, he again fixes the sheath to the instrument, and then withdraws it. By this method the internal incision is almost transverse, and forms a very obtuse angle with the external opening; but this angle may be readily effaced by pressing it with the finger, and presents no impediment to the introduction of the forceps, or the extraction of the stone. The author states that he has been in the habit of using the lithotome in this manner for the last ten years, and that he has never opened an artery large enough to produce any unpleasant hemorrhage.

*Second stage of the operation.*—After having made the requisite division of the prostate gland and orifice of the bladder, the surgeon introduces the left index-finger into the wound, in order to ascertain its extent, and be able to judge of the situation and size of the stone; this finger is also to be used to guide the forceps, unless the patient is very fat, and has a very enlarged prostate; in which circumstance it will be impossible to carry the finger into the bladder, and as it is then to be feared that the forceps will become entangled in the cellular tissue, and pass between the prostate and the rectum, it will be necessary to have recourse to another expedient. This consists in placing the fore-finger in the interior angle of the wound, and in applying the concavity of a gorget upon its radical edge, and introducing it into the bladder by pushing it from below upwards. When the gorget has arrived in the bladder, the finger is to be withdrawn, and the concavity of the instrument is to be brought upwards, while its convexity is to be made to rest slightly against the inferior angle of the wound. The surgeon then takes the forceps, and passing the index-finger along their blades, he makes them glide obliquely from below upwards, into the groove of the gorget, taking care that their convex surface shall correspond with the sides of the wound."

Bi-Lateral Operation.—This favorite operation of the Baron Dupuytren differs very little from that which has just been described, save that his lithotome is double. Two blades spring out from the trunk of the instrument, and are so used as to cut the prostate gland and membranous portion of the urethra on both sides, (whence the name bilateral). A greater space is thus obtained for the eduction of the calculus. In the hands of the distinguished operator I have just mentioned, this has proved a safe and certain mode. But I believe it has not spread very widely.

Recto-vesical Operation.—This operation was invented by Dr. L. J. Sanson. An attempt has been made to rob him of the honor, by stating that the operation had been described by an Italian in the 16th century, in a work published at Basle, under the name of Vegece, respecting whom the celebrated Haller uses the following words: "Jubet per vulnus recti intestini, et vesicæ aculeo lapidem ejicere." (*Bib. Chirurg., T. I.* p. 102). The operation, improved by Vacca Berlinghieri, is performed in the following manner, as described by Coster in Manual.

"The patient is placed as for the lateral operation: the staff is held perpendicularly by an assistant, the groove neither being turned to the right nor the left. The surgeon introduces the fore-finger of the left hand into the rectum, glides flatwise on the palmar face of this finger, the blade of a straight bistoury; and after having turned its edge upwards, he cuts by a single stroke from behind forwards, and in the direction of the raphe. This incision extends equally through about an inch of the intestine and raphe. The inferior surface of the prostate gland, being thus exposed, the finger easily perceives through the inconsiderable thickness of the parts, which separate the rectum and lower fundus of the bladder, which approach each other, the staff, which the assistant should always hold in the same position.

The surgeon passes the point of the bistoury behind the prostate, and again directs it from behind forwards, to make an incision through the prostate, urethra, and neck of the bladder, by carefully avoiding to injure its lower fundus. By this method the incision of the intestine will be found about an inch lower than the superior angle of the incision made through the neck of the bladder, and the walls of the rectum form a valve which prevents fecal matter from passing into the bladder.

The stone is then removed in the usual manner.

We neither introduce compress nor lint into the wound; and when the inflammation subsides, we touch the edges of the wound with lunar caustic, to hasten the cicatrization."

